# Long COVID-associated symptoms prevalent in both SARS-CoV-2 positive and negative individuals: A prospective follow-up study

**DOI:** 10.1016/j.nmni.2023.101209

**Published:** 2023-12-07

**Authors:** Anu Kantele, Juuso Paajanen, Jukka-Pekka Pietilä, Olli Vapalahti, Sari H. Pakkanen, Tinja Lääveri

**Affiliations:** a Meilahti Infectious Diseases and Vaccine Research Center, MeiVac, Biomedicum 1, Haartmaninkatu 8, Helsinki University Hospital and University of Helsinki, 00290, Helsinki, Finland; b Department of Infectious Diseases, Helsinki University Hospital, Helsinki, Finland; c Human Microbiome Research Program, Faculty of Medicine, Haartmaninkatu 4, 00014, University of Helsinki, Helsinki, Finland; d Department of Pulmonary Medicine, Heart and Lung Center, Helsinki University Hospital and University of Helsinki, Finland; e Viral Zoonoses Research Unit, Departments of Virology and Veterinary Biosciences, Faculties of Medicine and Veterinary Medicine, 00014, University of Helsinki, Helsinki, Finland; f Helsinki University Hospital Diagnostic Center, HUSLAB, Clinical Microbiology, Topeliuksenkatu 32, 00290, Helsinki, Finland; g Department of Computer Science, Aalto University, PO Box 15400, FI-00076, AALTO, Finland

**Keywords:** SARS-CoV-2, Long covid, Follow-up study

## Abstract

**Background:**

Research into persistent symptoms among SARS-CoV-2-positive i.e. CoV(+) patients mostly focuses on hospitalized individuals. Our prospective follow-up study compares long COVID-associated symptoms among laboratory-confirmed CoV(+) and SARS-CoV-2 negative [CoV(−)] individuals.

**Methods:**

SARS-CoV-2 RT-PCR-tested volunteers were recruited into four cohorts: 1) CoV(+) outpatients, 2) CoV(−) outpatients, 3) CoV(+) intensive care unit (ICU) inpatients, and 4) CoV(+) non-ICU inpatients. Neutralizing antibodies were assessed and questionnaires filled in at enrolment and days 90–120, 121–180, 181–270, 271–365, and 365–533.

**Results:**

Of the 1326 participants, 1191 were CoV(+): 46 ICU, 123 non-ICU, and 1022 outpatients; 135 were CoV(−) outpatient controls. Both CoV(+) outpatients and CoV(−) controls showed high overall symptom rates at all time points. More prevalent among CoV(+) than CoV(−) outpatients were only impaired olfaction and taste; many others proved more frequent for CoV(−) participants. At ≥181 days, fatigue, dyspnoea, various neuropsychological symptoms and several others were recorded more often for CoV(+) inpatients than outpatients.

**Conclusions:**

Long COVID-associated symptoms were more frequent among hospitalized than non-hospitalized CoV(+) participants. As for outpatients, only impaired olfaction and taste showed higher rates in the CoV(+) group; some symptoms proved even more common among those CoV(−). Besides suggesting low long COVID prevalences for outpatients, our results highlight the weight of negative controls.

## Highlights

• ‘Long COVID-associated symptoms’ were prevalent both among SARS-CoV-2 (+) and (−) participants.
• Only two symptoms – impaired olfaction and taste – proved more common in the SARS-CoV-2 (+) than the SARS-CoV-2 (−) group.
• Most of the others were more prevalent in the SARS-CoV-2 (−) than the SARS-CoV-2 (+) outpatient group.
• SARS-CoV-2 (+) inpatients had higher symptom rates than SARS-CoV-2 (+) outpatients.

## 1 Introduction

The coronavirus disease 2019 (COVID-19) caused by severe acute respiratory syndrome coronavirus 2 (SARS-CoV-2) has accounted for substantial morbidity and mortality worldwide. The clinical spectrum of COVID-19 ranges from asymptomatic to severe, life-threatening disease. Although most individuals with acute COVID-19 make full recovery, many studies – particularly the multitude conducted among hospitalized patients [[1], [2], [3], [4]] – describe for part of them persisting symptoms, referred to by such names as long COVID, post-COVID syndrome, or post-acute sequelae of SARS-CoV-2 infection [1].

While definitions of long COVID, its prevalence and range of manifestations vary, some symptoms, particularly fatigue, shortness of breath, smell and taste impairment, and cognitive dysfunction are commonly reported [1]. The prevalence of long COVID symptoms varies by research design from 75 % after hospital discharge [[1], [2], [3], [4]] to 10–30 % in population-based investigations [[5], [6], [7], [8], [9], [10], [11], [12], [13]]. Factors found to predispose to persistent symptoms include severity of disease, comorbidities, female sex, smoking, obesity, and increased age [1,13]. Longitudinal studies suggest a slow decrease in symptoms over time, with the majority still symptomatic after one year [14]. Prospective research looking at SARS-CoV-2 positive i.e. CoV(+) and SARS-CoV-2-negative i.e. CoV(−) outpatients remains a minority [[15], [16], [17], [18]]. Vaccines may provide protection and thus appear as a confounding factor [18,19]. Indeed, studies focusing on unvaccinated individuals are scarce.

Between February 2020 and April 2021, we recruited unvaccinated volunteers for a prospective follow-up cohort study comparing post-infective symptoms among COVID-19 outpatients and negative controls. While centring on outpatients, we also covered inpatients in the same design. Here, we report cross-sectional follow-up results from our symptom survey at five time points.

## 2 Materials and methods

We investigated long-term consequences of COVID-19 in a prospective cohort study in the HUS Helsinki University Hospital. Clearance was obtained from the Ethics Committee of HUS (HUS/1238/2020). All participants gave written informed consent. This study was conducted in accordance with the Declaration of Helsinki.

### 2.1 Study design and volunteers

We invited patients with a SARS-CoV-2 RT-PCR result in the HUS laboratory (HUSLAB) database to participate in the Clin-COVID master study exploring COVID-related symptoms and immune responses, contacting 20–50 first positives and 0–5 first negatives per week. At enrolment, volunteers provided blood samples and filled in questionnaires. They were informed about the opportunity to join a follow-up; the questionnaire links were sent later.

For the present long COVID study, we selected those recruited between February 29, 2020 and April 15, 2021 who had responded to at least one follow-up questionnaire >90 days after RT-PCR/symptom onset. The participants fell into four groups: 1) CoV(+) outpatients, 2) CoV(−) outpatients, 3) CoV(+) inpatients treated in an ICU; and 4) CoV(+) inpatients treated in a non-ICU ward. There were no restrictions concerning age, sex, nationality, or underlying illnesses. The data were retrieved in August 2021.

None of the participants had had COVID-19 or received SARS-CoV-2 vaccinations before their index RT-PCR test. All were tested for SARS-CoV-2 antibodies. Two with negative index RT-PCR but positive initial serology were included in the CoV(+) group.

If the participants contracted SARS-CoV-2 during the follow-up – as determined either by questionnaire data or, for CoV(−), also by a positive RT-PCR result in HUSLAB database – their data were included only until that point.

### 2.2 Collection of data

Electronic questionnaires (Webropol) used for data collection comprised background, baseline (Q0) and follow-up questionnaires grouped by follow-up time points of 90–120 days (Q120), 121–180 days (Q180), 181–270 days (Q270), 271–365 days (Q365), and over 365 (Q365+) days after symptoms onset/index RT-PCR test. Blood samples were collected 1–2 months after index RT-PCR test.

### 2.3 SARS-CoV-2-neutralizing antibodies (NAb)

In the microneutralization test (MNT) we used the Wuhan-like SARS-CoV Fin-1 strain on Vero E6-cells [20], defining titres ≥1:20 as positive.

### 2.4 Statistical analyses

In statistical analyses, we used SPSS v. 28.0 (IBM Corp., Armonk, NY). Two-tailed tests were employed with alpha level (significance) defined as P < 0.05. For categorical variables, the χ^2^-test, Fisher's exact test or binary logistic regression was used as appropriate, and for continuous variables the Mann-Whitney *U* test. For the mean geographic titres, SAS 9.4 (SAS Institute Inc., Cary, NC) was applied; the groups were compared using Kruskal-Wallis test by npar1way procedure with Wilcoxon option.

CoV(+) and CoV(−) outpatients were compared at all time points. For comparisons at >180 days, we combined the Q270, Q365 and Q365+ time points.

## 3 Results

### 3.1 Baseline characteristics

**Fig. 1:**
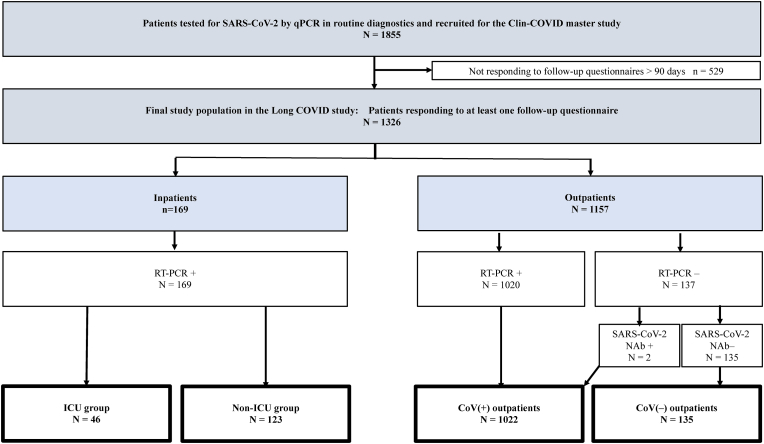
Flow chart of the study.

Of the 6666 patients initially invited to participate in the Clin-Covid master study, 1855 agreed to participate; 529 (28.5 ​%) did not return follow-up questionnaires. Among those initially invited, 18.5 ​% (135/730) of the RT-PCR negative and 20.1 ​% (1191/5936) RT-PCR positive returned at least one follow-up questionnaire at >3 months. The final study population comprised 1326 participants: 1022 in the CoV(+) outpatient and 135 in the CoV(−) outpatient group, and 46 in the CoV(+) ICU and 123 in the CoV(+) non-ICU ward group (Fig. 1 and Table 1). The median ages of the CoV(+) and CoV(−) outpatients were 47 and 46 years, with proportion of females 64 ​% and 79 ​%, respectively. Acute symptoms over the first two illness days were reported by 98 ​% of the CoV(+) outpatients and 84 ​% of the CoV(−) outpatients.

**Table 1:** Baseline characteristics of study participants.


Characteristics	Outpatients (n = 1157)		Hospitalized patients (n = 169)		Total (n = 1326)
	CoV (+) (n = 1022)	CoV (−) (n = 135)	CoV (+) non-ICU ward^a^ (n = 123)	CoV (+) ICU^b^ (n = 46)	CoV (+) (n = 1191)
Age, median (IQR)^c^	47.0 (35.0–57.0)	46.0 (36.0–57.0)	57.0 (47.0–65.0)	60.0 (52.5–64.5)	49.0 (37.0–59.0)
Sex, No (%)					
Female	655 (64)	107 (79)	63 (51)	21 (46)	739 (62)
Male	367 (36)	28 (21)	60 (49)	25 (54)	452 (38)
BMI, median (IQR)	25.2 (22.7–28.4)	25.3 (21.9–29.0)	28.1 (25.2–1.4)	29.6 (27.2–36.1)	25.6 (22.9–29.1)
Chronic disease, No (%)					
Diabetes	43 (6)	5 (5)	14 (17)	8 (19)	65 (8)
Asthma/COPD	90 (14)	19 (20)	25 (31)	9 (21)	124 (16)
Cardiovascular^d^	164 (25)	22 (23)	35 (43)	19 (44)	218 (28)
Rheumatoid arthritis	75 (11)	12 (13)	11 (14)	4 (9)	90 (11)
Hypothyroidism	24 (4)	5 (5)	3 (4)	1 (2)	28 (4)
Upper respiratory tract^e^	28 (4)	6 (6)	1 (1)	1 (2)	30 (4)
Cancer	24 (4)	4 (4)	7 (9)	6 (14)	37 (5)

### 3.2 SARS-CoV-2-neutralizing antibodies

In two-month blood samples, the geometric means of NAbs were 43 (95 ​% CI 39–47) for the CoV(+) outpatients, 99 (36–270) for the non-ICU, and 92 (34–245) for the ICU inpatients. A higher titre was found for those hospitalized (two groups combined) than the CoV(+) outpatients (P ​= ​0.0425).

### 3.3 Symptoms among CoV(+) and CoV(−) outpatients

The median (IQR) numbers of symptoms did not differ between the CoV(+) and CoV(−) outpatients (Supplementary Table 1). The five most common symptoms in the CoV(+) outpatient group were headache (31 ​%; range 27–36 ​% at the various time points), fatigue (28 ​%; 26–32 ​%), insomnia (24 ​%; 18–31 ​%), weakness or tiredness (23 ​%; 21–26 ​%), and joint symptoms (21 ​%; 18–26 ​%), and in the CoV(−) outpatient group headache (55 ​%; 49–67 ​%), fatigue (36 ​%; 28–46 ​%), rhinitis (34 ​%; 25–57 ​%), insomnia (32 ​%; 26–43 ​%), and lack of concentration (29 ​%; 24–43 ​%).

### 3.4 Comparison of symptoms between CoV(+) and CoV(−) outpatients

**Fig. 2:**
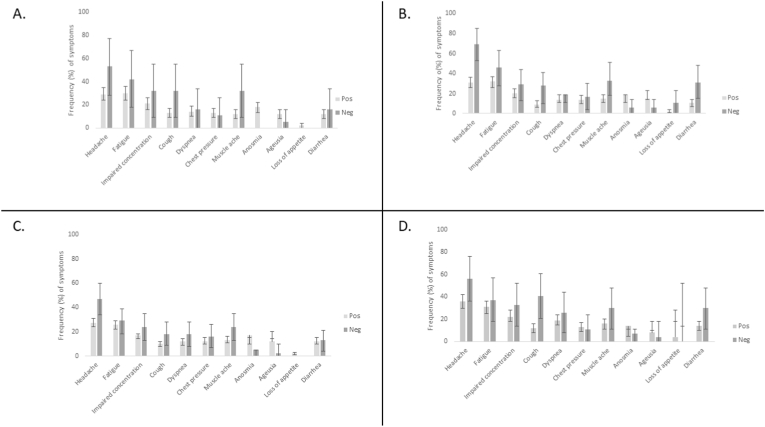
Percentage of CoV(+) and CoV(−) outpatients reporting selected symptoms at four timepoints. A. days 91–120; B. 121–180; C. 181–270; D. 271–365. Error bars are 95% confidence intervals.

At >180 days, 523 (74 ​%) in the CoV(+) and 70 (79 ​%) in the CoV(−) outpatient group reported at least one of the 42 symptoms in the questionnaire (P ​= ​0.320). Table 2 presents symptoms reported >180 days after the initial RT-PCR. For detailed symptom differences across time points, see Supplementary Table 1 and Fig. 2.

**Table 2:** Symptoms reported by CoV(+) and CoV(−) outpatients >180 days after initial symptom onset. ∗Fisher's exact test; all other tests were two-sided χ^2^-tests.

181 days or more	Total	CoV(+) outpatients	CoV(−) outpatients	P-value	OR (95 ​% CI) CoV(+) vs CoV(−)
	No (%)	No (%)	No (%)		
**Neurocognitive symptoms**					
Headache^a^	239 (30)	201 (28)	38 (48)	0.000	0.4 (0.3–0.7)
Vertigo/dizziness^a^	79 (10)	71 (10)	8 (10)	0.997	1.0 (0.5–2.2)
Fatigue^b^	207 (26)	181 (26)	26 (32)	0.225	0.7 (0.4–1.2)
Weakness/tiredness^b^	167 (21)	146 (21)	21 (26)	0.289	0.8 (0.4–1.3)
Numbness^b^	126 (16)	108 (15)	18 (22)	0.114	0.6 (0.4–1.1)
Sensory impairment^b^	97 (12)	86 (12)	11 (14)	0.734	0.9 (0.5–1.7)
Impaired concentration^b^	141 (18)	119 (17)	22 (27)	0.024	0.5 (0.3–0.9)
Impaired memory^b^	107 (14)	90 (13)	17 (21)	0.043	0.6 (0.3–1.0)
Difficulty in grasping the big picture^b^	74 (9)	59 (8)	15 (19)	0.003	0.4 (0.2–0.8)
Difficulties with oral expression^b^	25 (3)	19 (3)	6 (7)	0.036∗	0.3 (0.1–0.9)
Challenges in writing^b^	13 (2)	11 (2)	2 (2)	0.635∗	0.6 (0.1–2.9)
Reduced stress tolerance^b^	37 (5)	35 (5)	2 (2)	0.416∗	2.1 (0.5–8.8)
Irritability^b^	87 (11)	72 (10)	15 (19)	0.025	0.5 (0.3–0.9)
Melancholy^b^	110 (14)	93 (13)	17 (21)	0.058	0.6 (0.3–1.0)
Overly cheery mood^b^	67 (9)	49 (7)	18 (22)	0.000	0.3 (0.1–0.5)
Perceptual abnormality^b^	8 (1)	7 (1)	1 (1)	0.585∗	0.8 (0.1–6.6)
Insomnia^b^	162 (21)	136 (19)	26 (32)	0.008	0.5 (0.3–0.8)
Excessive nightmares^b^	77 (10)	63 (9)	14 (17)	0.018	0.5 (0.3–0.9)
Excessive sleepiness^b^	72 (9)	64 (9)	8 (10)	0.826∗	0.9 (0.4–2.0)
Excessive fears^b^	76 (10)	63 (9)	13 (16)	0.042	0.5 (0.3–1.0)
**Cardiorespiratory symptoms**					
Cough^a^	93 (12)	76 (11)	17 (21)	0.006	0.4 (0.2–0.8)
Rhinitis^a^	138 (17)	109 (15)	29 (36)	0.000	0.3 (0.2–0.5)
Dyspnoea^a^	100 (13)	85 (12)	15 (19)	0.085	0.6 (0.3–1.1)
Chest pressure^a^	101 (13)	91 (13)	10 (13)	0.932	1.0 (0.5–2.1)
Sore throat^a^	97 (12)	76 (11)	21 (26)	0.000	0.3 (0.2–0.6)
Ear pain^a^	28 (4)	23 (3)	5 (6)	0.192∗	0.5 (0.2–1.4)
**Gastrointestinal symptoms**					
Stomach ache^a^	80 (10	59 (8)	21 (26)	0.000	0.3 (0.1–0.4)
Flatulence^a^	108 (14)	87 (12)	21 (26)	0.001	0.4 (0.2–0.7)
Loose stools. diarrhoea^a^	98 (12)	83 (12)	15 (19)	0.070	0.6 (0.3–1.1)
Nausea^a^	47 (6)	35 (5)	12 (15)	0.001	0.3 (0.1–0.6)
**Olfaction and taste**					
Anosmia/impaired olfaction^a^	119 (15)	115 (16)	4 (5)	0.008	3.7 (1.3–10.3)
Dysgeusia/impaired taste^a^	81 (10)	79 (11)	2 (3)	0.016	4.9 (1.2–20.3)
**Other symptoms**					
Muscle ache^a^	114 (14)	93 (13)	21 (26)	0.002	0.4 (0.2–0.7)
Joint pain, swelling, stiffness^a^	164 (21)	144 (20)	20 (25)	0.327	0.8 (0.4–1.3)
Febrile feeling, fever^a^	27 (3)	22 (3)	5 (6)	0.182∗	0.5 (0.2–1.3)
Skin problems^a^	48 (6)	38 (5)	10 (13)	0.022	0.4 (0.2–0.8)
Eye pain^a^	41 (5)	33 (5)	8 (10)	0.057∗	0.4 (0.2–1.0)
Vision changes/blurry visions^a^	39 (5)	36 (5)	3 (4)	0.789∗	1.4 (0.4–4.6)
Weight loss^a^	14 (2)	12 (2)	2 (3)	0.644∗	0.7 (0.1–3.1)
Weight gain^a^	49 (6)	44 (6)	5 (6)	1.000∗	1.0 (0.4–2.6)
Loss of appetite^b^	12 (2)	12 (2)	0 (0)	0.624∗	NA
Increased appetite^b^	18 (2)	17 (2)	1 (1)	1.000∗	2.0 (0.3–15.1)

Total numbers of participants from whom data were collected are indicated by ^a^ and ^b^. Total number of responses in column “Total” is ^a^789 and ^b^782; CoV(+) outpatients column ^a^709 and ^b^701; CoV(−) outpatients column ^a^80 and ^b^81.

#### 3.4.1 Neurocognitive/CNS symptoms

At >180 days, many neurocognitive/CNS symptoms proved less prevalent in the CoV(+) than the CoV(−) group, including headache (28 ​%/48 ​%), concentration impairment (17 ​%/27 ​%), memory impairment (13 ​%/21 ​%), difficulty of perception (8 ​%/19 ​%), difficulties with oral expression (3 ​%/7 ​%), irritability (10 ​%/19 ​%), overly cheery mood (7 ​%/22 ​%), insomnia (19 ​%/32 ​%), excessive nightmares (9 ​%/17 ​%), and excessive fears (9 ​%/16 ​%). Differences for the other neurocognitive/CNS symptoms were minimal.

#### 3.4.2 Cardiorespiratory symptoms

Cough (11 ​%/21 ​%), rhinitis (15 ​%/36 ​%), and sore throat (11 ​%/26 ​%) were less frequent in the CoV(+) than the CoV(−) group at >180 days and most time points. For dyspnoea or chest pressure, no constant differences were noted.

#### 3.4.3 Gastrointestinal symptoms

Stomach ache (8 ​%/26 ​%), flatulence (12 ​%/26 ​%), and nausea (5 ​%/15 ​%) were less common among CoV(+) than CoV(−) individuals at >180 days, while loose stools were less common at two different time points (Q180 11 ​%/31 ​% and Q365 14 ​%/30 ​%).

#### 3.4.4 Olfaction and taste

Unlike the other symptoms, impaired olfaction proved more frequent in the CoV(+) than the CoV(−) group at >180 days (16 ​%/5 ​%, OR 3.7 (1.3–10.3) and most time points. Impaired taste also proved more common in the CoV(+) group at >180 days (11 ​%/3 ​%, OR 4.9 (1.2–20.3), yet with a significant difference only at one time point (Q270).

#### 3.4.5 Other symptoms

Myalgia was less frequent in the CoV(+) than the CoV(−) group at the three earliest time points and at >180 days (13 ​%/26 ​%). Skin problems (5 ​%/13 ​%) were more common at >180 days but not at individual time points. No significant differences were detected in the frequencies of joint symptoms, fever, eye symptoms, or changes of weight or appetite.

### 3.5 Comparisons between CoV(+) outpatients and ICU/non-ICU groups at >180 days

At >180 days, 523 (79 ​%) of the CoV(+) outpatients, 74 (78 ​%) of those in the non-ICU ward and 32 (97 ​%) in the ICU group reported at least one symptom (P ​= ​0.009; outpatients versus non-ICU ward P ​= ​0.388 and outpatients versus ICU P ​= ​0.017). The number of symptoms increased with the severity of SARS-CoV-2 infection: for the outpatients the median was 3 (IQR 1–7), for the non-ICU 4 (1–10), and the ICU group 8 (3–13) (P ​< ​0.001).

**Fig. 3:**
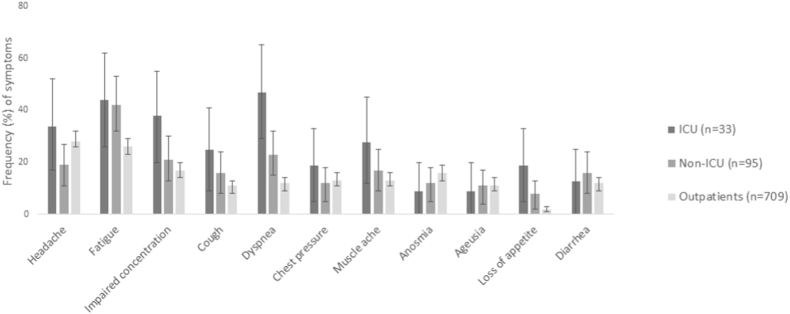
Percentage of CoV(+) patients reporting selected symptoms after 180 days from initial RT-PCR test. ICU, intensive care unit; Non-ICU, COVID-19 hospital ward.

Fig. 3 and Supplementary Table 2 show comparisons of symptoms between the CoV(+) outpatients and the non-ICU or ICU inpatients. Significant differences were observed for numerous neurocognitive/CNS symptoms: the respective proportions for fatigue were 26 ​%, 41 ​% and 44 ​%, for numbness 15 ​%, 20 ​% and 38 ​%, sensory impairment 12 ​%, 16 ​% and 28 ​%, impaired concentration 17 ​% 21 ​% and 38 ​%, excessive weakness/tiredness 21 ​%, 30 ​% and 38 ​%; difficulty in grasping the big picture 8 ​%, 14 ​% and 19 ​%; insomnia 19 ​%, 28 ​% and 50 ​%.

As for cardiorespiratory symptoms, among the CoV(+) outpatients cough (11 ​%) and dyspnoea (12 ​%) were less frequent than in the non-ICU (16 ​% and 23 ​%) and ICU (27 ​% and 48 ​%) groups.

Myalgia (13 ​%, 17 ​%, and 30 ​% for outpatient, non-ICU, and ICU groups, respectively), joint symptoms (20 ​%, 25 ​%, 45 ​%), and loss of appetite (19 ​%, 8 ​% and 2 ​%) were all reported more often by the inpatients than the CoV(+) outpatients.

### 3.6 Reinfections and new primary infections

Of CoV(+) and CoV (−) outpatients, respectively, 31 ​% (264/844) and 65 ​% (68/104) reported RT-PCR test taken during the follow-up (P ​< ​0.001; OR 0.2, 95 ​% CI 0.2–0.4). None of those in the CoV(+) and two in the CoV(−) group tested positive over the follow-up; review of the HUSLAB database revealed no additional positives in the CoV(−) group.

## 4 Discussion

Although COVID-19 is generally managed in outpatient settings, studies suggesting high incidences of long-term sequelae mainly look at hospitalized patients [[1], [2], [3], [4]]. However, CoV(−) control groups are missing in most reports, nor have they been set as inclusion criterion in the numerous reviews and meta-analyses [[2], [3], [4]]. Indeed, a recent cohort study involving CoV(+) outpatients and CoV(−) controls suggests for outpatients lower long COVID symptom frequencies [17] than those previously reported for inpatients. While restricting our prospective outpatient study to unvaccinated volunteers, our design also covered, so as to enable comparisons, three CoV(+) groups (outpatients and inpatients at wards or ICU) and CoV(−) controls.

Our results challenge most previous research: apart from impaired olfaction and taste, we found none of the long COVID-associated symptoms more common among the CoV(+) outpatients, but in fact several symptoms were reported more frequently by CoV(−) outpatients.

### 4.1 Symptoms among outpatients: which ones more common in CoV(+) than CoV(−) group?

More than two thirds of our CoV(+) outpatients reported at least one symptom, which accords with numerous other investigations [[5], [6], [7], [8], [9], [10], [11], [12],[14], [15], [16], [17], [18]]. The frequency of the most common symptoms among CoV(+) patients was slightly higher or in the same range as in previous outpatient studies: headache (27–36 ​% versus 0.4–14 ​% at various time points), fatigue (26–32 ​%/0.2–31 ​%), insomnia (18–31 ​%/2–10 ​%), weakness or tiredness (21–26 ​%/24–31 ​%), and joint symptoms (18–26 ​%/1–16 ​%) [[5], [6], [7], [8], [9], [10], [11], [12],[14], [15], [16], [17], [18]].

Somewhat unexpectedly our comparisons with the control group revealed only two symptoms to be more prevalent among CoV(+) than CoV(−) outpatients: impaired olfaction and taste. At 3–4 months the rates of impaired olfaction were 19 ​% in the CoV(+) and 5 ​% in the CoV(−) group and of impaired taste 15 ​% and 5 ​%, respectively; the differences were found throughout the follow-up. Other studies applying control groups also report respective rates [5,7,9,10,12,14,17,18,21]. Although seemingly trivial, these symptoms are associated with a lower quality of life [22].

Interestingly, none of the neurocognitive/CNS or cardiorespiratory symptoms considered characteristic of long COVID [1,17] proved more prevalent among our CoV(+) than our CoV(−) outpatients, contrasting findings of some previous outpatient research with control groups [5,10]. Why did our prospective study design not show higher rates for CoV(+) than CoV(−) outpatients? Some explanations appear logical: 1) High symptom frequencies reported for hospitalized patients do not apply to mild infections – the very low frequency among outpatients (resulting after subtracting rates in the CoV(−) control group) accords with some other researchers’ findings [[16], [17], [18]]. 2) Many long-term symptoms may not be COVID-19-specific – 84 % of our CoV(−) outpatients had respiratory tract symptoms at baseline – or not even related to the initial infection.

### 4.2 High symptom rates both in CoV(+) and CoV(−) outpatient groups

Including the CoV(−) control group proved critical: it not only revealed olfaction and taste to be the single symptoms with higher frequency among the CoV(+) than the CoV(−) groups, but also enabled us to demonstrate high overall symptom rates for all. The frequencies could be ascribed both to the prevailing pandemic involving various restrictions and pertaining problems, such as feelings of depression and anxiety [23] and fatigue [24], challenges in employment and economics [25], and loneliness [26]. Consistent with our data, recent studies [[16], [17], [18]] comparing CoV(+) and CoV(−) outpatients at three or six months, indicate high frequencies of post-infection type of symptoms for both groups. Furthermore, Deuel et al. reported that apart from some metabolic parameters, there were virtually no differences in long covid symptoms and findings between SARS-CoV2 antibody positive and negative Swiss army volunteers [27].

### 4.3 Several symptoms more common in CoV(−) than CoV(+) group

Our CoV(−) outpatients showed higher rates than our CoV(+) group for a number of symptoms, the data contradicting many prospective outpatient studies with negative controls [5,9,10,12,16,17], while according with some others [7,15]. Recently Gottlieb et al. reported higher prevalence of severe fatigue among CoV(–) than CoV(+) [18] patients.

The higher frequencies detected for the CoV(−) group might be explained simply by fear of getting infected which affected those not having contracted the disease as yet, entailing concern about even the mildest symptoms. Indeed, during the follow-up, RT-PCR tests were taken more frequently in the CoV(−) than the CoV(+) group. We found rather similar symptom rates among our SARS-CoV-2 seronegative healthcare workers at the onset of the pandemic in 2020 [28].

### 4.4 Symptom rates higher for CoV(+) in-than CoV(+) outpatients

Most of the studies with high post-infection sequelae rates solely look at hospitalized patients [[2], [3], [4]]. In our investigation, many typical long COVID symptoms (fatigue, impaired concentration, dyspnoea, weight loss) were also reported more frequently by CoV(+) inpatients than outpatients.

### 4.5 Serology

The CoV(−) outpatient status was confirmed by serological analyses. Consistent with previous studies [29], SARS-CoV-2-specific antibody levels proved higher among CoV(+) inpatients than outpatients.

## 5 Limitations

The CoV(−) group would have benefited from larger participant numbers. Of our CoV(−) outpatients, >80 % were symptomatic during/prior to the initial RT-PCR; an asymptomatic control group would also have been of interest. In addition, a CoV(−) inpatient group would have been valuable for distinguishing whether the higher symptom rate among inpatients was a result of COVID or simply post-infection sequelae following a severe disease. Indeed, severe infections have been associated with increased risk of neurological sequelae, such as dementia [30].

Our data does not cover delta and omicron variants which emerged in Finland after our recruitment [31]; long COVID appears less common after omicron infections [18,32].

Those with symptoms may have been more willing to respond – the same impact would be expected in both CoV(+) and CoV(−) groups.

While recruitment activity was not fully identical at all time points, all groups underwent a similar follow-up. Indeed, the lack of substantial changes in our cross-sectional prevalence data across the various time points aligns with the continued stress brought on by new emerging variants and lockdowns, affecting all participants alike. This result might have been different, had the pandemic subsided.

## 6 Strengths

Our major strengths include prospective research design, recruiting only unvaccinated volunteers, embracing a negative control group, and confirming it both by RT-PCR and serology. Our speedy start in the early days of the pandemic had major benefits: 1) the prevalence in Finland remained low for a long time, ensuring the controls a low SARS-CoV-2 risk over the follow-up [29] – indeed, only two CoV(−) outpatients contracted SARS-CoV-2. This contrasts to later population-based studies in which many of the matched control group members may have become positive without records in the national databases. 2) Thanks to our early start, all participants were unvaccinated at recruitment, eliminating the confounding protective effect of vaccines against long COVID [18,19].

## 7 Conclusions

The equally high frequencies of most long COVID-associated symptoms among CoV(−) and CoV(+) outpatients illustrate the need for control groups in research into postinfectious symptom prevalence. Here, the high rates also seen among the CoV(−) participants suggest that pandemic stress and anxiety may contribute to nonspecific symptoms. Rather than actually excluding the development of long COVID after mild disease, the data simply indicate a prevalence considerably lower than suggested in most previous studies.

## Ethical statement

The study protocol was approved by the Ethics Committee of HUS (HUS/1238/2020). All participants gave written informed consent.

## Funding

This study was supported by the 10.13039/501100003125Finnish Cultural Foundation, the Finnish Government Subsidy for Health Science Research (TYH2021315, TYH2021343), the 10.13039/100010135Finnish Medical Association, and the 10.13039/501100002341Academy of Finland (grant numbers 1336490, 336439 and 335527), HUS Inflammation Center Research Funds, Juho Vainio and Jane & Aatos Erkko Foundations. The funders of the study had no role in study design, data collection, data analysis, or writing of the report.

## CRediT authorship contribution statement

**Anu Kantele:** Conceptualization, Funding acquisition, Investigation, Methodology, Project administration, Resources, Supervision, Writing – original draft, Writing – review & editing. **Juuso Paajanen:** Conceptualization, Data curation, Investigation, Visualization, Writing – original draft, Writing – review & editing. **Jukka-Pekka Pietilä:** Data curation, Investigation, Methodology, Project administration, Writing – review & editing. **Olli Vapalahti:** Funding acquisition, Methodology, Resources, Supervision, Writing – review & editing. **Sari H. Pakkanen:** Data curation, Funding acquisition, Investigation, Project administration, Writing – review & editing. **Tinja Lääveri:** Conceptualization, Data curation, Formal analysis, Methodology, Software, Validation, Visualization, Writing – original draft.

## Declaration of competing interest

Tinja Lääveri: Honoraria (Pfizer) unrelated to this article. Anu Kantele: Research grants (Valneva, Pfizer) unrelated to this article. None of the authors declare any competing interests that could have influenced the work reported in this paper. Other authors declare no conflict of interests.
