# Supplementary material for: Long COVID-associated symptoms prevalent in both SARS-CoV-2 positive and negative individuals: A prospective follow-up study
Source: New Microbes New Infect. 2023 Dec 7;56:101209. doi: 10.1016/j.nmni.2023.101209 (PMC10761764; doi:10.1016/j.nmni.2023.101209)
Supplement: Multimedia component 1 [file mmc1.docx]

**Supplementary Table 1.** Symptoms reported by CoV(+) and CoV(–) outpatients. The data are given at different time points after initial onset of symptoms/ RT-PCR test. *Fisher’s exact test; all other tests were two-sided χ^2^-tests.

|  | **91–120 days (Q120)** | | | | **121–180 days (Q180)** | | | | **181–270 days (Q270)** | | | | **271–365 days (Q365)** | | | | **over 365 days (Q365+)** | | | |
| --- | --- | --- | --- | --- | --- | --- | --- | --- | --- | --- | --- | --- | --- | --- | --- | --- | --- | --- | --- | --- |
|  | CoV (+) | CoV (–) | P-value | OR  (95% CI) | CoV (+) | CoV (–) | P-value | OR (95% CI) | CoV (+) | CoV (–) | P-value | OR (95% CI) | CoV (+) | CoV (–) | P-value | OR (95% CI) | CoV (+) | CoV (–) | P-value | OR  (95% CI) |
|  | No (%) | No (%) |  |  | No (%) | No (%) |  |  | No (%) | No (%) |  |  | No (%) | No (%) |  |  | No (%) | No (%) |  |  |
| **Neurocognitive symptoms** |  |  |  |  |  |  |  |  |  |  |  |  |  |  |  |  |  |  |  |  |
| Headache^a^ | 72 (29) | 10 (53) | 0.035 | 0.4  (0.1–1.0) | 91 (31) | 25 (69) | 0.000 | 0.2  (0.1–0.4) | 175 (27) | 29 (47) | 0.001 | 0.4  (0.3–0.7) | 100 (36) | 16 (56) | 0.050 | 0.5  (0.2–1.0) | 60 (36) | 4 (57) | 0.426* | 0.4  (0.1–2.0) |
| Vertigo/  dizziness^a^ | 26 (11) | 1  (5) | 0.703* | 2.1  (0.3–16.7) | 35 (12) | 3 (8) | 0.781* | 1.5  (0.4–5.0) | 65 (10) | 6 (10) | 0.948* | 1.0  (0.4–2.5) | 29 (11) | 4 (15) | 0.515* | 0.7  (0.2–2.1) | 18 (11) | 2 (29) | 0.187* | 0.3  (0.1–1.7) |
| Fatigue^b^ | 73 (30) | 8 (42) | 0.262 | 0.6  (0.2–1.5) | 93 (32) | 16 (46) | 0.092 | 0.5  (0.3–1.1) | 163 (26) | 18 (29) | 0.596 | 0.9  (0.5–1.5) | 85 (31) | 10 (37) | 0.505 | 0.8  (0.3–1.7) | 47 (28) | 2 (29) | 1.000* | 1.0  (0.2–5.3) |
| Weakness/  tiredness^b^ | 57 (23) | 5 (26) | 0.780* | 0.8  (0.3–2.5) | 78 (26) | 13 (37) | 0.180 | 0.6  (0.3–1.3) | 132 (21) | 16 (25) | 0.393 | 0.8  (0.4–1.4) | 63 (23) | 6 (22) | 0.943 | 1.0  (0.4–2.7) | 42 (25) | 2 (29) | 1.000* | 0.8  (0.2–4.5) |
| Numbness^b^ | 43 (18) | 5 (26) | 0.355* | 0.6  (0.2–1.7) | 46 (16) | 8 (23) | 0.272 | 0.6  (0.3–1.5) | 95 (15) | 14 (22) | 0.130 | 0.6  (0.3–1.2) | 53 (19) | 6 (22) | 0.705 | 0.8  (0.3–2.2) | 39 (23) | 2 (29) | 0.670* | 0.8  (0.1–4.1) |
| Sensory impairment^b^ | 29 (12) | 3 (16) | 0.712* | 0.7  (0.2–2.6) | 34 (12) | 5 (14) | 0.584* | 0.8  (0.3–2.2) | 75 (12) | 7 (11) | 0.869 | 1.1  (0.5–2.4) | 36 (13) | 6 (22) | 0.237* | 0.5  (0.2–1.4) | 21 (13) | 1 (14) | 1.000* | 0.9  (0.1–7.6) |
| Impaired concentration^b^ | 52 (21) | 6 (32) | 0.386* | 0.6  (0.2–1.6) | 60 (20) | 10 (29) | 0.260 | 0.6  (0.3–1.4) | 107 (17) | 15 (24) | 0.165 | 0.6  (0.4–1.2) | 62 (22) | 9 (33) | 0.203 | 0.6  (0.2–1.4) | 41 (25 | 3 (43) | 0.372* | 0.4  (0.1–2.0) |
| Impaired memory^b^ | 38 (16) | 3 (16) | 1.000* | 1.0  (0.3–3.5) | 43 (15) | 5 (14) | 0.963 | 1.0  (0.4–2.8) | 78 (12) | 12 (19) | 0.127 | 0.6  (0.3–1.2) | 43 (16) | 6 (22) | 0.409* | 0.6  (0.2–1.7) | 26 (16) | 3 (43) | 0.093* | 0.2  (0.1–1.2) |
| Difficulty in perception of the overall picture^b^ | 29 (12) | 3 (16) | 0.712* | 0.7  (0.2–2.6) | 35 (12) | 6 (17) | 0.413* | 0.7  (0.3–1.7) | 52 (8) | 10 (16) | 0.041 | 0.5  (0.2–1.0) | 41 (15) | 7 (26) | 0.163* | 0.5  (0.2–1.3) | 29 (17) | 3 (43) | 0.119* | 0.3  (0.1–1.3) |
| Difficulties with oral expression^b^ | 14  (6) | 1 (5) | 1.000* | 1.1  (0.1–8.8) | 10 (3) | 2 (6) | 0.370* | 0.6  (0.1–2.8) | 17 (3) | 4 (6) | 0.112* | 0.4  (0.1–1.2) | 12 (4) | 2 (7) | 0.360* | 0.6  (0.1–2.7) | 4 (2) | 1 (14) | 0.189* | 0.1  (0.0–1.5) |
| Challenges in writing^b^ | 6  (2) | 0  (0) | 1.000* | NA | 6 (2) | 1 (3) | 0.547* | 0.7  (0.1–6.0) | 9 (1) | 2 (3) | 0.261* | 0.4  (0.1–2.1) | 11 (4) | 1 (4) | 1.000* | 1.1  (0.1–8.7) | 8 (5) | 0 (0) | 1.000* | NA |
| Reduced stress tolerance^b^ | 7  (3) | 0  (0) | 1.000* | NA | 7 (2) | 8 (23) | 0.000* | 0.1  (0.0–0.2) | 34 (5) | 2 (3) | 0.763* | 1.7  (0.4–7.4) | 4 (2) | 1 (4) | 0.432* | 0.5  (0.1–4.3) | 4 (2) | 1 (14) | 0.189* | 0.1  (0.0–1.5) |
| Irritability^b^ | 36 (15) | 2 (11) | 1.000* | 1.5  (0.3–6.6) | 33 (11) | 12 (34) | 0.001* | 0.2  (0.1–0.5) | 66 (10) | 13 (21) | 0.014 | 0.4  (0.2–0.9) | 28 (10) | 5 (19) | 0.193* | 0.5  (0.2–1.4) | 17 (10) | 3 (43) | 0.034* | 0.2  (0.0–0.7) |
| Melancholy^b^ | 44 (18) | 3 (16) | 1.000* | 1.2  (0.3–4.2) | 44 (15) | 12 (34) | 0.004 | 0.3  (0.2–0.7) | 80 (13) | 14 (22) | 0.033 | 0.5  (0.3–1.0) | 47 (17) | 6 (22) | 0.594* | 0.7  (0.3–1.9) | 20 (12) | 2 (29) | 0.219* | 0.3  (0.1–1.9) |
| Cheerful mood^b^ | 44 (18) | 4 (21) | 0.758* | 0.8  (0.3–2.6) | 37 (13) | 4 (11) | 1.000* | 1.1  (0.4–3.3) | 40 (6) | 14 (22) | 0.000* | 0.2  (0.1–0.5) | 43 (16) | 7 (26) | 0.176* | 0.5  (0.2–1.3) | 21 (13) | 2 (29) | 0.234* | 0.4  (0.1–2.0) |
| Perceptual abnormality^b^ | 3 (1) | 1 (5) | 0.260* | 0.2  (0.0–2.3) | 5 (2) | 0 (0) | 1.000* | NA | 6 (1) | 0 (0) | 1.000* | NA | 1 (0) | 1 (4) | 0.171* | 0.1  (0.0–1.6) | 2 (1) | 0 (0) | 1.000* | NA |
| Insomnia^b^ | 65 (27) | 5 (26) | 0.984 | 1.0  (0.4–2.9) | 72 (24) | 12 (34) | 0.205 | 0.6  (0.3–1.3) | 115 (18) | 18 (29) | 0.044 | 0.6  (0.3–1.0) | 86 (31) | 10 (37) | 0.531 | 0.8  (0.3–1.7) | 51 (31) | 3 (43) | 0.679* | 0.6  (0.1–2.7) |
| Excessive nightmares^b^ | 40 (16) | 2 (11) | 0.747* | 1.7  (0.4–7.5) | 40 (14) | 3 (9) | 0.596* | 1.7  (0.5–5.7) | 53 (8) | 10 (16) | 0.047 | 0.5  (0.2–1.0) | 45 (16) | 5 (19) | 0.786* | 0.9  (0.3–2.4) | 29 (17) | 2 (29) | 0.610* | 0.5  (0.1–2.9) |
| Excessive sleepiness^b^ | 8 (3) | 0 (0) | 1.000* | NA | 24 (8) | 5 (14) | 0.213* | 0.5  (0.2–1.5) | 58 (9) | 6 (10) | 0.919 | 1.0  (0.4–2.3) | 15 (5) | 3 (11) | 0.208* | 0.5  (0.1–1.7) | 2 (1) | 1 (14) | 0.117* | 0.1  (0.0–0.9) |
| Excessive fears^b^ | 29 (12) | 3 (16) | 0.712* | 0.7  (0.2–2.6) | 36 (12) | 7 (20) | 0.190* | 0.6  (0.2–1.4) | 48 (8) | 10 (16) | 0.023 | 0.4  (0.2–0.9) | 48 (17) | 3 (11) | 0.591* | 1.7 (0.5–5.8) | 27 (16) | 1 (14) | 1.000* | 1.2  (0.1–10.1) |
| **Cardiorespiratory symptoms** |  |  |  |  |  |  |  |  |  |  |  |  |  |  |  |  |  |  |  |  |
| Cough^a^ | 32 (13) | 6 (32) | 0.036* | 0.3  (0.1–0.9) | 27 (9) | 10 (28) | 0.003* | 0.3  (0.1–0.6) | 65 (10) | 11 (18) | 0.063 | 0.5  (0.3–1.0) | 34 (12) | 11 (41) | 0.001* | 0.2  (0.1–0.5) | 25 (15) | 3 (43) | 0.085* | 0.2  (0.0–1.1) |
| Rhinitis^a^ | 52 (21) | 6 (32) | 0.386* | 0.6  (0.2–1.6) | 49 (16) | 9 (25) | 0.200 | 0.6  (0.3–1.3) | 92 (14) | 20 (32) | 0.000 | 0.4  (0.2–0.6) | 66 (24) | 12 (44) | 0.021 | 0.4  (0.2–0.9) | 30 (18) | 4 (57) | 0.028* | 0.2  (0.0–0.8) |
| Dyspnea^a^ | 35 (14) | 3 (16) | 0.743* | 0.9  (0.2–3.2) | 43 (14) | 7 (19) | 0.426 | 0.7  (0.3–1.7) | 75 (12) | 11 (18) | 0.161 | 0.6  (0.3–1.2) | 53 (19) | 7 (26) | 0.408 | 0.7  (0.3–1.7) | 29 (17) | 3 (43) | 0.119* | 0.3  (0.1–1.3) |
| Chest pressure^a^ | 32 (13) | 2 (11) | 1.000* | 1.3  (0.3–5.8) | 42 (14) | 6 (17) | 0.678 | 0.8  (0.3–2.1) | 80 (12) | 10 (16) | 0.403 | 0.7  (0.4–1.5) | 35 (13) | 3 (11) | 1.000* | 1.2  (0.3–4.1) | 13 (8) | 3 (43) | 0.018* | 0.1  (0.0–0.6) |
| Sore throat^a^ | 21 (9) | 6 (32) | 0.007* | 0.2  (0.1–0.6) | 35 (12) | 13 (36) | 0.000 | 0.2  (0.1–0.5) | 65 (10) | 17 (27) | 0.000 | 0.3  (0.2–0.5) | 29 (11) | 6 (22) | 0.106* | 0.4  (0.2–1.1) | 21 (13) | 1 (14) | 1.000* | 0.9  (0.1–7.6) |
| Ear pain^a^ | 9 (4) | 2 (11) | 0.183* | 0.3  (0.1–1.6) | 10 (3) | 2 (6) | 0.626* | 0.6  (0.1–2.8) | 20 (3) | 5 (8) | 0.060* | 0.4  (0.1–1.0) | 15 (5) | 1 (4) | 1.000* | 1.5  (0.2–11.8) | 6 (4) | 2 (29) | 0.035* | 0.1  (0.0–0.6) |
| **Gastrointestinal symptoms** |  |  |  |  |  |  |  |  |  |  |  |  |  |  |  |  |  |  |  |  |
| Stomach ache^a^ | 25 (10) | 4 (21) | 0.141* | 0.4  (0.1–1.4) | 32 (11) | 5 (14) | 0.574* | 0.7  (0.3–2.1) | 47 (7) | 14 (23) | 0.000 | 0.3  (0.1–0.5) | 35 (13) | 8 (30) | 0.037* | 0.3  (0.1–0.9) | 21 (13) | 2 (29) | 0.234* | 0.4  (0.1–2.0) |
| Flatulence^a^ | 39 (16) | 7 (37) | 0.030* | 0.3  (0.1–0.9) | 48 (16) | 11 (31) | 0.032 | 0.4  (0.2–0.9) | 79 (12) | 14 (23) | 0.022 | 0.5  (0.3–0.9) | 56 (20) | 9 (33) | 0.118 | 0.5  (0.2–1.2) | 32 (19) | 1 (14) | 1.000* | 1.4  (0.2–12.3) |
| Loose stools, diarrhea^a^ | 30 (12) | 3 (16) | 0.716* | 0.7  (0.2–2.7) | 33 (11) | 11 (31) | 0.003* | 0.3  (0.1–0.6) | 77 (12) | 8 (13) | 0.827 | 0.9  (0.4–2.0) | 39 (14) | 8 (30) | 0.048* | 0.4  (0.2–1.0) | 23 (14) | 1 (14) | 1.000* | 1.0  (0.1–8.4) |
| Nausea^a^ | 14 (6) | 3 (16) | 0.113* | 0.3  (0.1–1.2) | 16 (5) | 3 (8) | 0.444* | 0.6  (0.2–2.3) | 31 (5) | 9 (15) | 0.005* | 0.3  (0.1–0.7) | 14 (5) | 4 (15) | 0.065* | 0.3  (0.1–1.0) | 4 (2) | 2 (29) | 0.020* | 0.1  (0.0–0.4) |
| **Olfaction and taste** |  |  |  |  |  |  |  |  |  |  |  |  |  |  |  |  |  |  |  |  |
| Anosmia/  impaired olfaction^a^ | 43 (18) | 0  (0) | 0.050* | NA | 56 (19) | 2 (6) | 0.048 | 3.9  (0.9–16.9) | 106 (17) | 3 (5) | 0.014 | 4.0  (1.2–12.9) | 38 (14) | 2 (7) | 0.552* | 2.0  (0.5–8.8) | 23 (14) | 1 (14) | 1.000* | 1.0  (0.1–8.4) |
| Ageusia/  impaired taste^a^ | 30 (12) | 1  (5) | 0.709* | 2.5  (0.3–19.5) | 46 (15) | 2 (6) | 0.110 | 3.1  (0.7–13.4) | 76 (12) | 1 (2) | 0.014 | 8.2  (1.1–59.7) | 22 (8) | 1 (4) | 0.706* | 2.3  (0.3–17.5) | 14 (8) | 0 (0) | 1.000* | NA |
| **Other symptoms** |  |  |  |  |  |  |  |  |  |  |  |  |  |  |  |  |  |  |  |  |
| Muscle ache^a^ | 29 (12) | 6 (32) | 0.026* | 0.3  (0.1–0.8) | 44 (15) | 12 (33) | 0.005 | 0.3  (0.2–0.7) | 82 (13) | 15 (24) | 0.012 | 0.5  (0.2–0.9) | 43 (16) | 8 (30) | 0.100* | 0.4  (0.2–1.1) | 37 (22) | 2 (29) | 0.656* | 0.7  (0.1–3.8) |
| Joint pain, swelling, stiffness^a^ | 44 (18) | 6 (32) | 0.218* | 0.5  (0.2–1.3) | 64 (21) | 12 (33) | 0.109 | 0.5  (0.3–1.2) | 133 (21) | 12 (19) | 0.809 | 1.1  (0.6–2.1) | 60 (22) | 10 (37) | 0.074 | 0.5  (0.2–1.1) | 43 (26) | 1 (14) | 0.680* | 2.1  (0.2–17.9) |
| Febrile feeling, fever^a^ | 7  (3) | 2 (11) | 0.130* | 0.3  (0.0–1.3) | 6 (2) | 2 (6) | 0.209* | 0.3  (0.1–1.8) | 19 (3) | 4 (6) | 0.135* | 0.4  (0.1–1.3) | 12 (4) | 1 (4) | 1.000* | 1.2  (0.1–9.5) | 5 (3) | 2 (29) | 0.027* | 0.1  (0.0–0.5) |
| Skin problems^a^ | 20 (8) | 2 (11) | 0.664* | 0.8  (0.2–3.5) | 20 (7) | 2 (6) | 1.000* | 1.2  (0.3–5.5) | 37 (6) | 7 (11) | 0.096* | 0.5  (0.2–1.1) | 14 (5) | 4 (15) | 0.065* | 0.3  (0.1–1.0) | 12 (7) | 1 (14) | 0.427* | 0.5  (0.1–4.2) |
| Eye pain^a^ | 10 (4) | 1 (5) | 0.568* | 0.8  (0.1–6.3) | 14 (5) | 2 (6) | 0.686* | 0.8  (0.2–3.8) | 27 (4) | 5 (8) | 0.190* | 0.5  (0.2–1.3) | 15 (5) | 3 (11) | 0.210* | 0.5  (0.1–1.7) | 10 (6) | 2 (29) | 0.076* | 0.2  (0.0–0.9) |
| Vision changes/blurry visions^a^ | 9 (4) | 0 (0) | 1.000* | NA | 13 (4) | 0 (0) | 0.375* | NA | 32 (5) | 3 (5) | 1.000* | 1.0  (0.3–3.5) | 14 (5) | 1 (4) | 1.000* | 1.4  (0.2–11.0) | 15 (9) | 2 (29) | 0.142* | 0.2  (0.0–1.4) |
| Weight loss^a^ | 4 (2) | 1 (5) | 0.314* | 0.3  (0.0–2.8) | 7 (2) | 1 (3) | 0.603* | 0.8  (0.1–7.0) | 11 (2) | 2 (3) | 0.285* | 0.5  (0.1–2.2) | 8 (3) | 0 (0) | 1.000* | NA | 7 (4) | 0 (0) | 1.000* | NA |
| Weight gain^a^ | 7 (3) | 0 (0) | 1.000* | NA | 25 (8) | 2 (6) | 0.752* | 1.6  (0.4–6.9) | 42 (7) | 4 (6) | 1.000* | 1.0  (0.4–2.9) | 32 (12) | 2 (7) | 0.751* | 1.6  (0.4–7.3) | 11 (7) | 1 (14) | 0.401* | 0.4  (0.0–3.9) |
| Loss of appetite^a^ | 5 (2) | 0 (0) | 1.000* | NA | 7 (2) | 4 (11) | 0.021* | 0.2  (0.1–0.7) | 11 (2) | 0 (0) | 0.611* | NA | 10 (4) | 0 (0) | 0.608* | NA | 7 (4) | 0 (0) | 1.000* | NA |
| Increased appetite^a^ | 8 (3) | 2 (11) | 0.156* | 0.3  (0.1–1.5) | 11 (4) | 0 (0) | 0.614* | NA | 14 (2) | 1 (2) | 1.000* | 1.4  (0.2–10.8) | 10 (4) | 0 (0) | 0.608* | NA | 3 (2) | 1 (14) | 0.154* | 0.1  (0.0–1.2) |
|  | **91–120 days (Q120)** | | | | **121–180 days (Q180)** | | | | **181–270 days (Q270)** | | | | **271–365 days (Q365)** | | | | **over 365 days (Q365+)** | | | |
|  | CoV (+) | CoV (–) | P-value | OR  (95% CI) | CoV (+) | CoV (–) | P-value | OR  (95% CI) | CoV (+) | CoV (–) | P-value | OR  (95% CI) | CoV (+) | CoV (–) | P-value | OR  (95% CI) | CoV (+) | CoV (–) | P-value | OR  (95% CI) |
| **Number of symptoms, medium (IQR)** | 2.0  (1­–7) | 4.5  (2–11) | 0.170 | NA | 3.0  (1–7) | 2.0  (0–10) | 0.137 | NA | 3.0  (1–7) | 3.0  (1–9) | 0.868 | NA | 4.0  (1–9) | 5.0  (0–8) | 0.317 | NA | 4.0  (1–9) | 6.5  (1–19) | 0.764 | NA |

The total number of participants from whom the data were collected are indicated by ^a^ and ^b^. The total number of responses in column “91–120 days” is for CoV(+) ^a^245 and ^b^245, for CoV(–) ^a^19 and ^b^19; “121–180 days” for CoV(+) ^a^298 and ^b^295, for CoV(–) ^a^36 and ^b^35; “181–270 days” CoV(+) ^a^644 and ^b^635, for CoV(–) ^a^62 and ^b^63; “271–365 days” CoV(+) ^a^275 and ^b^276, CoV(–) ^a^27 and ^b^27; “over 365 days” CoV(+) ^a^166 and ^b^166, CoV(–) ^a^7 and ^b^7

**Supplementary Table 2.** Symptoms reported by CoV(+) patients initially treated in ICU, hospital ward or as outpatients > 180 days after the initial onset of symptoms/RT-PCR test. Comparisons with ICU-treated patients were conducted with binary logistic regression analysis

|  | **ICU** | **Non-ICU** | **Outpatient** | **P-value** | **OR (95% CI)**  **Outpatient vs. non-ICU** | **OR (95% CI)**  **Outpatient vs. ICU** |
| --- | --- | --- | --- | --- | --- | --- |
|  | No (%) | No (%) | No (%) |  |  |  |
| **Neurocognitive symptoms** |  |  |  |  |  |  |
| Headache^a^ | 12 (36) | 18 (19) | 201 (28) | 0.081 | 1.0 (0.6–1.6) | 0.7 (0.3–1.4) |
| Vertigo/dizziness^a^ | 8 (24) | 11 (12) | 71 (10) | 0.035 | 1.1 (0.5–2.2) | 0.3 (0.2–0.8) |
| Fatigue^b^ | 14 (44) | 40 (41) | 181 (26) | 0.001 | 1.2 (0.7–2.1) | 0.4 (0.2–0.9) |
| Weakness/tiredness^b^ | 12 (38) | 29 (30) | 146 (21) | 0.018 | 1.3 (0.7–2.2) | 0.4 (0.2–0.9) |
| Numbness^b^ | 12 (38) | 20 (20) | 108 (15) | 0.003 | 1.5 (0.8–3.0) | 0.3 (0.1–0.6) |
| Sensory impairment^b^ | 9 (28) | 16 (16) | 86 (12) | 0.024 | 1.3 (0.6–2.7) | 0.4 (0.2–0.8) |
| Impaired concentration^b^ | 12 (38) | 21 (21) | 119 (17) | 0.009 | 1.0 (0.6–1.8) | 0.3 (0.2–0.7) |
| Impaired memory^b^ | 8 (25) | 13 (13) | 90 (13) | 0.141 | 0.9 (0.5–1.7) | 0.4 (0.2–1.0) |
| Difficulty in perception of the overall picture^b^ | 6 (19) | 14 (14) | 59 (8) | 0.034 | 3.0 (0.9–9.7) | 0.4 (0.2–1.0) |
| Difficulties with oral expression^b^ | 2 (6) | 4 (4) | 19 (3) | 0.416 | NA | 0.4 (0.1–1.9) |
| Challenges in writing^b^ | 2 (6) | 2 (2) | 11 (2) | 0.148 | NA | 0.2 (0.1–1.1) |
| Reduced stress tolerance^b^ | 4 (13) | 5 (5) | 35 (5) | 0.178 | 0.8 (0.3–1.9) | 0.4 (0.1–1.1) |
| Irritability^b^ | 7 (22) | 15 (15) | 72 (10) | 0.053 | 1.9 (0.8–4.5) | 0.4 (0.2–1.0) |
| Melancholy^b^ | 8 (25) | 18 (18) | 93 (13) | 0.086 | 1.0 (0.5–2.0) | 0.5 (0.2–1.1) |
| Cheerful mood^b^ | 2 (6) | 15 (15) | 49 (7) | 0.016 | 1.3 (0.5–3.4) | 1.1 (0.3–4.9) |
| Perceptual abnormality^b^ | 0 (0) | 0 (0) | 7 (1) | 0.520 | 0.7 (0.1–6.1) | NA |
| Insomnia^b^ | 16 (50) | 27 (28) | 136 (19) | 0.000 | 0.9 (0.5–1.6) | 0.2 (0.1–0.5) |
| Excessive nightmares^b^ | 7 (22) | 9 (9) | 63 (9) | 0.052 | 1.4 (0.6–3.3) | 0.4 (0.1–0.8) |
| Excessive sleepiness^b^ | 7 (22) | 11 (11) | 64 (9) | 0.054 | 1.8 (0.7–4.6) | 0.4 (0.1–0.9) |
| Excessive fears^b^ | 7 (22) | 11 (11) | 63 (9) | 0.048 | 1.3 (0.6–2.9) | 0.4 (0.1–0.8) |
| **Cardiorespiratory symptoms** |  |  |  |  |  |  |
| Cough^a^ | 9 (27) | 15 (16) | 76 (11) | 0.008 | 0.9 (0.4–1.7) | 0.3 (0.1–0.7) |
| Rhinitis^a^ | 7 (21) | 15 (16) | 109 (15) | 0.665 | 1.3 (0.7–2.6) | 0.7 (0.3–1.6) |
| Dyspnea^a^ | 16 (48) | 22 (23) | 85 (12) | 0.000 | 1.5 (0.7–3.3) | 0.1 (0.1–0.3) |
| Chest pressure^a^ | 6 (18) | 11 (12) | 91 (13) | 0.616 | 0.9 (0.5–1.6) | 0.7 (0.3–1.6) |
| Sore throat^a^ | 4 (12) | 11 (12) | 76 (11) | 0.942 | 1.5 (0.7–3.4) | 0.9 (0.3–2.5) |
| Ear pain^a^ | 1 (3) | 4 (4) | 23 (3) | 0.881 | 0.5 (0.2–1.4) | 1.1 (0.1–8.2) |
| **Gastrointestinal symptoms** |  |  |  |  |  |  |
| Stomach ache^a^ | 4 (12) | 11 (12) | 59 (8) | 0.458 | 0.9 (0.4–2.0) | 0.7 (0.2–1.9) |
| Flatulence^a^ | 5 (15) | 18 (19) | 87 (12) | 0.183 | 1.3 (0.6–2.8) | 0.8 (0.3–2.1) |
| Loose stools, diarrhea^a^ | 4 (12) | 15 (16) | 83 (12) | 0.521 | 2.0 (0.8–4.7) | 1.0 (0.3–2.8) |
| Nausea^a^ | 2 (6) | 5 (5) | 35 (5) | 0.953 | 1.2 (0.4–3.4) | 0.8 (0.2–3.5) |
| **Olfaction and taste** |  |  |  |  |  |  |
| Anosmia/impaired olfaction^a^ | 3 (9) | 11 (12) | 115 (16) | 0.296 | 0.6 (0.4–1.1) | 1.9 (0.6–6.5) |
| Ageusia/impaired taste^a^ | 3 (9) | 10 (11) | 79 (11) | 0.923 | 1.1 (0.5–2.3) | 1.3 (0.4–4.2) |
| **Other symptoms** |  |  |  |  |  |  |
| Muscle ache^a^ | 10 (30) | 16 (17) | 93 (13) | 0.016 | 0.9 (0.5–1.7) | 0.3 (0.2–0.8) |
| Joint pain, swelling, stiffness^a^ | 15 (45) | 24 (25) | 144 (20) | 0.002 | 1.5 (0.8–2.8) | 0.3 (0.2–0.6) |
| Febrile feeling, fever^a^ | 3 (9) | 2 (2) | 22 (3) | 0.132 | NA | 0.3 (0.1–1.1) |
| Skin problems^a^ | 3 (9) | 8 (8) | 38 (5) | 0.354 | 2.6 (0.6–10.9) | 0.6 (0.2–1.9) |
| Eye pain^a^ | 1 (3) | 6 (6) | 33 (5) | 0.691 | 0.6 (0.3–1.4) | 1.6 (0.2–11.8) |
| Vision changes/blurry visions^a^ | 4 (12) | 7 (7) | 36 (5) | 0.168 | 1.8 (0.5–6.0) | 0.4 (0.1–1.2) |
| Weight loss^a^ | 3 (9) | 2 (2) | 12 (2) | 0.013 | 0.7 (0.2–3.3) | 0.2 (0.0–0.6) |
| Weight gain^a^ | 5 (15) | 9 (9) | 44 (6) | 0.083 | 0.9 (0.4–2.0) | 0.4 (0.1–1.0) |
| Loss of appetite^b^ | 6 (19) | 8 (8) | 12 (2) | 0.000 | 0.8 (0.2–3.9) | 0.1 (0.0–0.2) |
| Increased appetite^b^ | 1 (3) | 4 (4) | 17 (2) | 0.623 | 0.5 (0.2–1.6) | 0.8 (0.1–6.0) |

The total numbers of participants from whom the data were collected are indicated by ^a^ and ^b^. The total number of responses in column “ICU” is ^a^33 and ^b^32; “non-ICU” ^a^95 and ^b^98; “outpatients” ^a^709 and ^b^701;
